# Distress detection in VR environment using Empatica E4 wristband and Bittium Faros 360

**DOI:** 10.3389/fphys.2025.1480018

**Published:** 2025-03-05

**Authors:** Jelena Medarević, Nadica Miljković, Kristina Stojmenova Pečečnik, Jaka Sodnik

**Affiliations:** ^1^ Faculty of Electrical Engineering, University of Ljubljana, Ljubljana, Slovenia; ^2^ School of Electrical Engineering, University of Belgrade, Belgrade, Serbia

**Keywords:** virtual reality, user experience, wearables, Empatica E4, Faros 360, distress detection, mean heart rate, RMSSD

## Abstract

**Introduction:**

Distress detection in virtual reality systems offers a wealth of opportunities to improve user experiences and enhance therapeutic practices by catering to individual physiological and emotional states.

**Methods:**

This study evaluates the performance of two wearable devices, the Empatica E4 wristband and the Faros 360, in detecting distress in a motion-controlled interactive virtual reality environment. Subjects were exposed to a baseline measurement and two VR scenes, one non-interactive and one interactive, involving problem-solving and distractors. Heart rate measurements from both devices, including mean heart rate, root mean square of successive differences, and subject-specific thresholds, were utilized to explore distress intensity and frequency.

**Results:**

Both the Faros and E4 sensors adequately captured physiological signals, with Faros demonstrating a higher signal-to-noise ratio and consistency. While correlation coefficients were moderately positive between Faros and E4 data, indicating a linear relationship, small mean absolute error and root mean square error values suggested good agreement in measuring heart rate. Analysis of distress occurrence during the interactive scene revealed that both devices detect more high- and medium-level distress occurrences compared to the non-interactive scene.

**Discussion:**

Device-specific factors in distress detection were emphasized due to differences in detected distress events between devices.

## 1 Introduction

Virtual Reality (VR) environments have gained significant popularity in recent years, offering immersive and interactive experiences that can simulate realistic scenarios. Alongside the visual and auditory components, the measurement within VR environments can provide a deeper understanding of human responses and experiences. By capturing physiological signals such as heart rate (HR), electrodermal activity (EDA), and motion data (MD) like acceleration, researchers can explore the correlations of user engagement, emotional states, cognitive processes, and user experiences during VR interactions (Egan et al., 2016). VR’s ability to create a strong sensation of being physically present in the virtual environment and the perception that virtual events are genuinely occurring ensures that users react to virtual scenarios as they would in real-life and collectively contribute to overall sense of presence in virtual environment (Slater et al., 2022).

The information collected with distress detection in VR systems has the potential to enhance user experience and holds promising implications across various fields. In VR therapy, it can be used to monitor and regulate patients’ emotional states during exposure sessions (Rahman et al., 2022), such as in the treatment of phobias (Raghav et al., 2016) or Post-Traumatic Stress Disorder (PTSD) (Wout et al., 2017). Physiological monitoring systems, detecting indicators like increased heart rate (Rahman et al., 2022) and skin conductance (Wout et al., 2017), allow therapists to dynamically adjust virtual environments in real-time, optimizing therapy based on individual needs. In training simulations, particularly in high-pressure scenarios such as medical (Rahman et al., 2022) or military (Wout et al., 2017) training, distress detection becomes a valuable tool. By evaluating trainees’ stress levels, VR systems can identify areas requiring additional support or practice (Parsons and Reinebold, 2012). Beyond therapy and training, distress detection contributes significantly to human-computer interaction, enabling VR systems to adapt the presented content based on the users’ emotional states for more natural and intuitive interactions (Duric et al., 2002). Additionally, VR systems equipped with physiological sensors can offer stress management experiences, providing guided meditation or calming environments that respond to users’ distress levels, creating a feedback loop to enhance relaxation (Gromala et al., 2015). The field of Neuroergonomics, which examines brain function in real-world environments, offers another potential application of distress detection in VR, particularly in optimizing user-performance in safety-critical professions (Parasuraman, 2003).

One of the primary physiological signals used for distress detection is heart rate, which measures the number of heart beats per minute. Heightened distress or emotional responses can lead to changes in HR (Mack et al., 2006), making it a fundamental parameter in assessing distress levels during VR experiences (Robitaille and McGuffin, 2019). Recent research suggests that there might be subtle differences in HR patterns between males and females. Studies have indicated that females tend to exhibit slightly higher average resting HRs compared to males, which could be attributed to hormonal and physiological variations between the sexes (Altini and Plews, 2021; Quer et al., 2020). Beyond sex-related distinctions, HR is influenced by a variety of factors, including physical activity levels, stressors, emotional states (Wu et al., 2019), fatigue (Tran et al., 2009), and even caffeine consumption (Koenig et al., 2013) and environmental conditions (Tiwari et al., 2021).

For reliable distress detection, the non-intrusivity of measurement devices is paramount. In VR, user immersion and experience are crucial, and intrusive devices can compromise data accuracy and user comfort. Wearable sensors and cameras provide non-intrusive data acquisition (Heikenfeld et al., 2018), preserving the naturalness of the VR experience and encouraging user compliance. In this context, we decided to utilize Empatica E4 (E4) wristband and Faros 360 chest strap (Faros), particularly as in the previous research we already validated E4 against Faros (Gruden et al., 2019). This study focused on evaluating E4 and Faros 360 devices in assessing drivers’ physiological responses during various driving conditions, emphasizing their effectiveness in measuring heart rate variability (HRV) and EDA, but noting challenges with motion artifacts affecting data quality, particularly in distinguishing different driving demands. However, it showed that the user-friendly nature of E4 sets it apart in experimental settings, offering easy mounting and usage—crucial factors when subjects are engaged in multitasking scenarios requiring sustained focus. Such non-intrusive nature of E4 wristband ensures seamless data collection (Heikenfeld et al., 2018) contributing to the authenticity and reliability of physiological responses in virtual reality settings.

However, it is important to note that results of (McCarthy et al., 2016) point out the low data quality of physiological signals obtained using E4 due to motion artefacts, especially the Blood Volume Pulse (BVP) signal, often used to estimate the HR signal. Even though Empatica released a new device – (Empatica Embrace Plus, 2024), we chose E4 device as its data is easily accessible (Looff et al., 2022), since the new device does not provide the API or access to the raw data in real time anymore. With the new device the data collection should be performed through a proprietary Empatica app and web server which is not ideal for research purposes, but it is worth noting that, for offline purposes, researchers can access raw.*avro* files from the server, and if needed, convert it to *.csv* format (Béquet, 2023). Furthermore, E4 device is still present on the market and majority of researchers still use it. One of Faros’ main limitations is direct-skin placement, which may be uncomfortable for some subjects, especially those with skin sensitivities or those requiring prolonged wear. Adhesive reactions, pressure from the chest strap, and sweating can impact user comfort and compliance. Its placement makes it less practical in applied settings like workplace monitoring, and its requirement to have precise sensor placement adds to setup complexity and potentially affects data quality.

Through the E4 measurement evaluation and comparison with Faros, this paper explores the importance of reliable data acquisition in VR environments for distress detection. Our proposed method for distress detection involves a straightforward thresholding approach and a rule-based system, contributing to the precision and efficiency of the analysis. The method uses distress detection thresholds that are subject-specific in order to tailor the method to each subject’s unique physiological profile.

Subjects were exposed to a baseline measurement and two VR scenes–a non-interactive scene (NIS) in which the subjects observed nature, and an interactive scene (IS) with distress induction in which the subjects were required to solve the Hanoi tower problem using a VR controller while being surprised with various distractors.

The main research questions addressed in this study are as follows:

1. Can both the Faros and E4 devices effectively detect distress in individuals in IS?
2. What is the level of data quality (determined through level of noise contamination) achieved by the E4 device when measuring heart rate used for distress detection?

The first research question explores firstly the possibility of using Faros/E4 in distress intensity and frequency detection based on the HR parameters, and secondly also the E4 performance compared to Faros. Only responses collected in the IS are considered since this scene is created for the purpose of eliciting distress in test subjects.

The second question is primarily focused on validating the performance and data quality of the E4 device assessed by evaluating the level of noise present in the heart rate measurements used for distress detection. The goal is to confirm if E4 can emerge as a user-friendly option for future studies, by exploring whether its heart rate measurements exhibit sufficiently high data quality, ensuring that distress detection remains unaffected. Baseline measurements, NIS and IS data is considered, since it is expected that the data quality is high for each measurement.

In summary, these research questions form the core inquiries guiding the investigation, aiming to assess the accuracy and potential of the E4 device and the comparative performance of the Faros and E4 devices in distress detection.

## 2 Materials and methods

In this section, the experimental design and procedures used to investigate physiological responses in a motion-controlled virtual environment are outlined. Two commercially available devices, Empatica E4 wristband and Faros, which were utilized to measure and record various physiological parameters during the study, are introduced. Before the experiment description, an overview of each device’s capabilities and functionalities is provided, setting the context for their use in this research. Subsequently, the experiment design is presented, along with subject details, and the VR scenes employed to capture physiological data.

### 2.1 Empatica E4 wristband device

**FIGURE 1:**
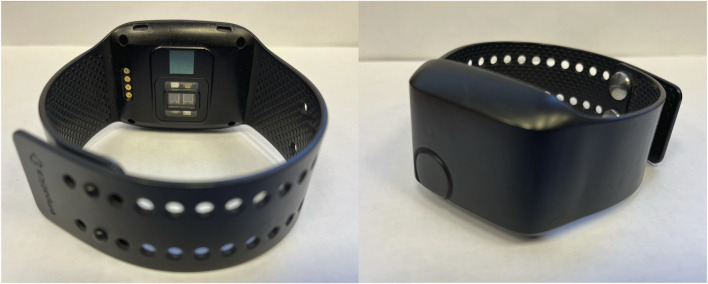
Empatica E4 device. This photography was taken at the Faculty of Electrical Engineering, University of Ljubljana.

Empatica E4 wristband (shown in Figure 1) is a commercially available physiological monitoring device (Empatica, 2024). It is equipped with several sensors that enable the measurement of multiple physiological parameters. Using photoplethysmography (PPG) it can capture the Blood Volume Pulse, which provides information on changes in blood volume in the microvascular bed, allowing for the estimation of HR and inter-beat interval (IBI) (Allen, 2007). Additionally, the device includes an EDA sensor, which measures changes in the electrical conductance of the skin, reflecting the user’s sympathetic nervous system activity and emotional responses (Boucsein, 2012). Moreover, the E4 wristband incorporates a temperature sensor, enabling the monitoring of skin temperature variations, a 3-axis accelerometer that measures acceleration in three directions, enabling the detection of motion and physical activity that can help researchers understand the subjects’ movements and activity levels during data collection.

Utilizing E4 wristband within a motion-controlled VR environment offers several advantages, particularly in the context of capturing physiological responses. The devices’ less obtrusive and user-friendly nature allows seamless data collection without disrupting subjects’ experiences in the VR scenarios. Being worn on the wrist, it enables continuous monitoring and real-time data transmission, making it well-suited for prolonged data collection during immersive VR sessions.

However, it is crucial to address potential limitations associated with the E4 wristband. Subjects’ activities during VR sessions may introduce motion artifacts and affect data quality. To mitigate this issue, careful measures were taken to control subjects’ motion during data collection, ensuring more accurate and reliable physiological measurements (Böttcher et al., 2022). Considering the context of our study, the E4 wristband ease of use, portability, and compatibility with VR scenarios make it an appropriate choice for HR.

### 2.2 Faros 360 device

**FIGURE 2:**
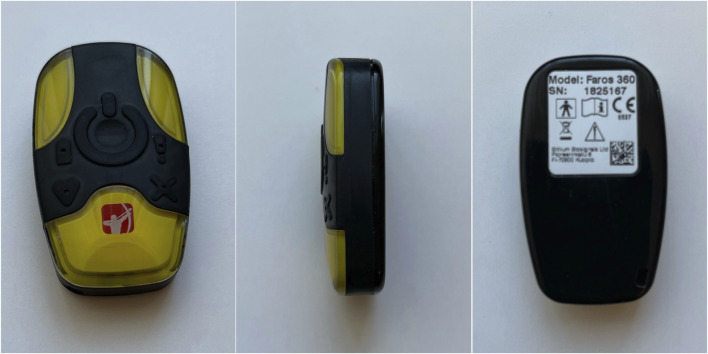
Bittium Faros 360 device. This photography was taken at the Faculty of Electrical Engineering, University of Ljubljana.

Faros 360 (shown in Figure 2) is a commercially available physiological monitoring device designed for electrocardiographic (ECG) signal recording. It allows the measurement of the electrical activity of the heart, providing information on heart rate and cardiac rhythm. The device is equipped with high-quality ECG sensors that enable accurate and reliable data collection, and enables 3-channels ECG measurement and data streaming via Bluetooth (Bittium Faros, 2024).

This device focuses on ECG measurements and high-quality ECG signals which makes it well-suited for providing precise HR data, crucial in understanding subjects’ cardiovascular responses during VR scenes in a motion-controlled VR environment.

However, it is essential to consider the limitations associated with Faros application in the VR context. The device is not capable of capturing other physiological parameters, such as EDA or skin temperature, which can also provide important information about subjects’ emotional and physiological states during VR experiences. Additionally, the placement of ECG electrodes on the chest may introduce potential challenges, as subjects may have to wear additional equipment that could affect their comfort and immersion during the VR sessions. There are three different ways to mount Faros to a participant’s chest, including Fast-Fix (Bittium’s proprietary electrode), cable sets, and using a textile belt with two electrodes and a mounting pad for Faros. For our study, we chose the third option, using a textile belt with two electrodes and a mounting pad, to balance signal quality and participant comfort. Faros 360 was chosen for this study due to its specialization in ECG measurements, allowing the acquisition of precise HR data during the motion-controlled VR scenes. By leveraging the capabilities of Faros 360, subjects’ cardiac responses can be understood, enhancing insights into their physiological reactions. Moreover, Faros 360 serves as a valuable reference for evaluating the performance of E4 wristband. Through a comparison of the data obtained from both devices, the consistency and reliability of the E4 wristband physiological measurements in the VR environment can be assessed. This comparative analysis has the potential to provide a comprehensive understanding of the strengths and limitations of each device, enabling informed decisions about their applications in future physiological research within VR settings.

### 2.3 Virtual reality

The study was conducted with the HTC Vive Pro Eye (2024) VR Headset (HTC Corporation, Vive, 2024). The system consists of a headset with integrated glasses with stereoscopic screens for displaying content in virtual reality, and two hand-held controllers that are used to manipulate and interact with the environment and the displayed objects in it. The two screens (one for each eye) of the glasses are high-definition OLED screens with a diagonal of 8.89 cm (3.5 inches). Each screen has a resolution of 1440 × 1600 pixels, which means that the headset displays content with a total resolution of 2880 × 1600 pixels or 615 pixels per inch. The refresh rate is up to 90 Hz and offers 110-degree field of view. The headset straps and the distance between the screens are adjustable, which allows for adaptations that best conform to the subjects’ needs (head size, pupillary distance, etc.). The hand-held controllers have a touch-sensitive surface, which the subject uses to input controls in a similar way as they would when using a touchpad on a laptop computer. The headset is equipped with speakers for playing sound.

For the baseline measurement subjects were equipped with Faros and E4 wristband for measuring the HR and BVP (respectively) and seated quietly with no significant movement, on a chair in the middle of the cabinet for 4 minutes. The length of the baseline data capture was consistent with the second and third parts of the experiment to ensure uniformity across all phases. This initial phase provided data on participants’ resting state and physiological responses in the absence of virtual reality stimuli.

In the second part of the experiment, participants remained seated with both Faros and E4, but this time they also wore HTC Vive Pro Eye VR headset. The basic scene of Steam VR Home was used as the non-interactive scene (NIS), which is a virtual environment consisting of a house with a terrace on top of a mountain. The house is surrounded by trees, and in the distance the subject has a beautiful view of the surrounding mountains. In the background, the subject can hear the wind blowing and birds chirping. During the experiment, the subjects were placed on the mentioned terrace, where they could observe the view in the distance and the birds flying around them. This environment was chosen with a goal to keep the subjects calm during this scene and to not induce distress.

The interactive VR scene (IS) was created using Blender (Stichting Blender Foundation, Amsterdam, Netherlands) for the visual elements, and Unity (Unity Software Inc., San Francisco, United States) for the creation of the actual scene and implementation of the Hanoi Tower problem game. The scene was set in a poorly lit and slightly dimmed warehouse. A forklift is driving in the background and ambient sound of a warehouse and the forklift moving is played through the speakers. Shelves with cardboard boxes are placed left and right from the subject.

**FIGURE 3:**
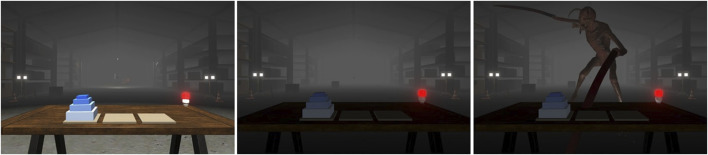
Left) Fall of the first box, center) fall of the second box, right) monster attack.

The subject is (virtually) seated in front of a table in the middle of the room (Figure 3), so they cannot see any of the walls of the space. A table lamp is lit red at the beginning of the test, which turns green upon successful completion of the Hanoi Tower task.

The scene test starts when the subject is satisfied with the placement of the VR and the view (and distance) they have of the table in front, which enables easy and comfortable moving of the cubes to complete the task. The subject is instructed to only move the dominant hand, keeping the non-dominant hand on the armrest. On the table, bases and cubes with different sizes are presented for solving the Hanoi Tower problem. The subject is first presented with three cubes and asked to arrange them in a predefined pattern. Upon successful completion, the scene ends. The scene is then reset, and the subject is presented with an additional cube, resulting in four cubes. Again, the subject is asked to arrange them in a predefined pattern. Upon successful completion, the scene ends. After that, the last scene is presented, where the subject is presented with five cubes and again asked to arrange them in a predefined pattern. Two minutes (120 s) after the start of the scene, one of the boxes falls from the left shelf to the floor accompanied by a loud bang. A few seconds before the end of the 3 minutes (180 s), a tension sound effect like typical sounds used in horror movies is played. This effect adds an extra level of suspense by telling the test taker that something is about to happen. As soon as the tension sound effect ends, another box falls from the right shelving unit, this time with a louder bang and further into the room. Unlike the first event, the second fall causes the lights on the ceiling to flicker, which can also be heard, for 1.5 s. Boxes falling are very short events, lasting less than 5 s.

After 4 minutes (240 s), a monster, making loud noises, jumps from the ceiling in front of the test subject and starts attacking them. As the monster lands on the ground, the lights in the background go out and the warehouse becomes very dark. A light placed under the table and aimed at the monster begins to flicker, illuminating the monster’s face. This lasts less than 5 s, and at this point, the scene and the whole trial ends. The subject is at that moment instructed to take off the VR glasses.

### 2.4 Experiment design

The study involved subjects aged over 18 and under 40, due to the difference in physiological signals after a certain age (Quer et al., 2020; Zhang, 2007; Acharya et al., 2004), with no known cardiovascular diseases. Each subject participated on a voluntary basis, and they could withdraw or stop the experiment at any point. The study was conducted in accordance with Declaration of Helsinki (General Assembly of the World Medical Association, 2014) and the study design as well as the study execution strictly followed the Code of ethics for researchers and Guidelines for ethical conduct in research involving people issued by (University of Ljubljana, 2024). Before the study, the participants were informed about the study goals and asked to sign the Informed consent provided by the ethical committee of University of Ljubljana. We acquired data on the subjects’ sex referring to the biological features related to both physical and physiological characteristics (Coen and Banister, 2012). The information on participant’s sex was self-reported on a voluntary basis.

We have followed Cohen’s guidelines for interpreting effect sizes (Cohen, 1988), with a slight modification for effect size distribution analysis for HRV studies as suggested by (Quintana, 2016; Laborde et al., 2017). The data for three subjects was not recorded appropriately in total, so the final sample size resulted in 8 female and 10 male subjects participated in the study, with a mean age of 22.3 ± 1.3 years (minimum age: 20, maximum age: 25).

The experiment was conducted in a motion-controlled virtual reality environment. Prior to the experiment, both the E4 and Faros devices were attached to the subject. Subjects were seated, with the E4 device positioned on their non-dominant hand, which they were instructed to keep on an armrest throughout the experiment to minimize movement. They were also instructed on the importance of remaining still to ensure data quality. Interaction with the VR scene was conducted using their dominant hand. Faros was placed with the textile belt right below the chest muscle. Once the VR headset was turned on, physiological responses were measured using both devices simultaneously. Each subject was exposed to a:

1. Baseline measurement
2. Non-interactive scene (NIS)
3. Interactive scene (IS),

and for each condition a 4-min recording was obtained.

### 2.5 Variables

In order to perform data quality assessment and distress detection, several key metrics had to be calculated.

#### 2.5.1 Data quality metrics

The Signal-to-Noise Ratio (SNR) was estimated for each heart rate signal, measuring the strength of the desired signal relative to background noise or interference (Box, 1988). Psignal ​ was calculated as the average of squared HR values recorded by each device, and Pnoise ​ was estimated as the average squared difference between the HR values and their mean–essentially the variance of the HR signal. This calculation was performed separately for both the Empatica and Faros devices.

$$SNR=10\log_{10}\frac{P_{signal}}{P_{noise}}$$

Higher SNR values indicate stronger and more reliable heart rate signals, providing insights into signal fidelity and measurement accuracy.

Correlation is a statistical method used to evaluate the potential linear relationship between two continuous variables. The correlation coefficient, a dimensionless quantity ranging from −1 to +1, quantifies the strength of this presumed linear association. A coefficient closer to +1 indicates a strong positive correlation, while a value closer to −1 suggests a strong negative correlation. A coefficient near 0 indicates a weak or no linear relationship between the variables (Swinscow and Campbell, 2002; Witte R.S. and Witte J.S., 2017). In this study, both Pearson (Kirch, 2008) and Spearman (Dodge, 2008) correlation coefficients were employed to assess the linear and monotonic relationships between the Faros and E4 HR signals, respectively. While Pearson correlation measures linear associations, Spearman correlation evaluates monotonic relationships, making it less sensitive to nonlinear associations or outliers (Siegel and Castellan, 1981).

Furthermore, the Mean Absolute Error (MAE) and Root Mean Square Error (RMSE) (Willmott et al., 1985) were computed for both Faros and E4 HR signals for each subject. The MAE represents the average absolute difference between corresponding heart rate values from Faros and E4, with Faros considered the reference or “ground truth” value. A smaller MAE indicates a lower overall error, reflecting a higher level of agreement between the two devices. On the other hand, RMSE represents the square root of the averaged squared differences between heart rate values, placing greater emphasis on larger errors compared to MAE.

Each of these metrics was calculated for 16 out of 18 subjects’ baseline, NIS and IS scene, excluding two subjects with corrupted signals in IS. Out of the remaining 16 subjects, six were female and 10 were male.

#### 2.5.2 Distress metrics

To compare the distress detection capabilities of E4 wristband and Faros based on HR features, two parameters were used:

1. Mean Heart Rate (Mean HR) provides an average HR value over a specific time period and serves as a measure of central tendency for HR data. It can be useful for understanding the overall level of cardiac activity during a specific time interval. It is commonly used to compare HR values between individuals or different conditions, such as rest and exercise (Karvonen and Vuorimaa, 1988). Mean HR is typically expressed in beats per minute [bpm].
2. Root Mean Square of Successive Differences (RMSSD) on the other hand, quantifies the variability in time intervals between consecutive heartbeats. It is calculated by taking the square root of the average of the squared differences between adjacent HR values. RMSSD reflects the high-frequency components of HRV, which are primarily influenced by parasympathetic (vagal) activity (Shaffer and Ginsberg, 2017). Higher RMSSD values indicate greater variability in HR, suggesting a more flexible autonomic nervous system and better cardiac health. RMSSD is often used as a marker of parasympathetic activity and can be used to assess the balance between sympathetic and parasympathetic regulation of the heart.

As outlined in (Stauss, 2014), it is crucial to avoid using HRV parameters in isolation without considering the mean level of HR, as this approach can lead to serious misinterpretation of experimental data. In this study, we chose Mean HR and RMSSD since their accuracy is preserved even when short-term recordings are used (Baek et al., 2015). Also, these two features are both important parameters in the analysis of HRV and are commonly used in research and clinical settings to assess autonomic function, cardiac health, and the impact of interventions or conditions on the cardiovascular system (Kamath et al., 2012).

Ultra-short-term (UST) recordings for HRV estimation have shown promise due to their efficiency in clinical and research settings. While UST measurements exhibit strong correlations with longer recordings for certain HRV parameters, such as mean HR and RMSSD, their accuracy may vary for other parameters like standard deviation of NN intervals (SDNN). Contextual factors, such as recording method (e.g., ECG vs PPG), age, health and artifact removal procedures, and the choice of HRV parameters can influence the reliability of UST measurements (Shaffer and Ginsberg, 2017). In this study, a 10 s window for segmentation and calculating mean HR and RMSSD was employed, since the correlations with the longer short-term recordings was reported as high enough–for mean HR in (Baek et al., 2015; Shaffer et al., 2016) and for RMSSD in (Baek et al., 2015; Nussinovitch et al., 2011; Salahuddin et al., 2007). Also, using a time window of 10 s helps capture short-term fluctuations in heart rate. It provides a balance between capturing immediate changes and avoiding noise or transient spikes that may occur within shorter time intervals. Munoz et al. (2015) conducted a comprehensive investigation into HRV measurements across a large adult sample (N = 3,387) and found that averaging over multiple 10-s segments, regardless of whether they are continuous, can provide reliable estimates of RMSSD. This approach aligns with contemporary practices in HRV analysis, where shorter recording periods are deemed sufficient for capturing meaningful physiological variability in quasi real-time. However, further research is needed to standardize protocols, establish normative values, and ensure consistent application of UST HRV measurements in place of conventional 5-min and 24-h recordings (Electrophysiology, 1996).

### 2.6 Data analysis

The data analysis section focuses on the processing and evaluation of the physiological data collected from both the E4 wristband and the Faros device during the motion-controlled VR experiment. The data undergoes thorough preprocessing to ensure data quality, followed by an assessment of E4 data quality. The section further explores distress detection using data from both devices and examines distress intensity and frequency. Additionally, a detailed analysis of distress during the interactive scene is conducted to gain insights into subjects’ physiological responses.

#### 2.6.1 Preprocessing

The collected physiological signals underwent comprehensive preprocessing to improve data quality and reliability. For this step and the analysis, Python version 3.9.7 (Python Software Foundation, Delaware, United States) was used. Synchronization between physiological recordings and VR events was achieved using timestamps from both data sources, since the distress events timestamps were known. The Faros ECG signal, sampled at 500 Hz, was subjected to various preprocessing steps using the *biosppy* Python package (Carreiras et al., 2018) and its *ecg.py* script. The first step was the application of a bandpass Finite Impulse Response (FIR) filter to eliminate artifacts outside of ECG frequency range. The order of this filter was calculated as 0.3*sampling rate, and the cutoff frequencies were set to 3 and 45 Hz, with a goal to eliminate baseline drift, remove low-frequency noise such as muscle artifacts and electrode motion artifacts, preserve ECG waveform and exclude higher-frequency noise. Hamilton segmentation (Hamilton, 2002) was used to accurately detect and isolate the QRS complexes and correction of R-peaks was done using template matching. Heart rate was calculated based on the array of R-peaks, and it was smoothed using moving average filter of type boxcar and window size equal to three samples (6 ms).

Similarly, the E4 BVP signal, sampled at 64 Hz, underwent preprocessing using the *ppg.py* script of the *biosspy* package. The first preprocessing step for the BVP signal involved filtering using 4th Butterworth bandpass filter with cutoff frequencies set at 1 and 8 Hz, applied with a goal to remove respiration influence (0.2–0.33 Hz), high frequency noise, and preserve heart rate range (from 1 Hz to 3 Hz, or 60–180 bpm). Both filters from *ppg.py* and *ecg.py* scripts use *filtfilt* function from the *scipy* package to perform zero-phase filtering, meaning that the filter is applied in both the forward and reverse directions, effectively eliminating any phase distortion introduced by the filtering process.

Onset detection was performed utilizing Elgendi’s method (Elgendi et al., 2013), and heartbeat extraction was done using detected peaks. The final HR signals were obtained using moving average smoothing of type *boxcar* and window size set to three samples (46.88 ms).

Both HR signals were upsampled to 4 Hz, to ensure accurate alignment between the two datasets, facilitating meaningful comparative analysis of the physiological responses captured by the Faros and E4 devices.

#### 2.6.2 Data quality assessment

To ensure the quality and reliability of the HR signals, key metrics described in Section 2.5.1. were calculated. SNR was used to evaluate each signal, with higher SNR values being preferable.

The correlation coefficient was used to quantify the similarity between the Faros and E4 HR signals, providing insights into their relationship. A higher correlation indicates a stronger connection and similar patterns, validating the accuracy and reliability of E4 HR measurements compared to Faros as the reference. A strong correlation signifies good E4 signal quality and reliability, while lower correlation may suggest potential measurement errors or artifacts.

MAE and RMSE were compared in order to determine the level of agreement between E4 and Faros for each of the conditions, with lower values indicating a higher degree of agreement.

In addition to the calculated metrics, Bland-Altman plot (Altman and Bland, 1983), a statistical method for assessing the agreement between two measurement techniques, was generated, and analyzed to further assess the agreement between Faros and E4 HR signals. This plot provides a comprehensive visualization of the mean differences and limits of agreement, offering valuable insights into the overall consistency and potential bias between the two measurement methods, aiding in the identification of any systematic bias or trends that may not be apparent in individual metrics.

#### 2.6.3 Distress detection using Faros and E4: intensity and frequency

In order to detect distress during both scenes, three additional preprocessing steps needed to be performed: HR signal segmentation, HR feature calculation and feature threshold calculation, after which the detection analysis was performed.

HR signal was segmented using a 10 s window, and features were calculated as described in Section 2.5.2. The calculation of feature thresholds was guided by the recognition that heart rate can vary significantly among individuals due to factors such as sex, age, fitness level, health conditions, and other physiological differences (Alexandre et al., 2012). To account for these individual variations (Whitehead et al., 1977), a personalized threshold approach was implemented, aiming to establish distinct thresholds for each subject based on their unique baseline HR.

The baseline measurement served as the reference for deriving feature thresholds to compare with signal segments from NIS and IS. For the Mean HR feature, the threshold was determined by setting it to the minimum value of each subject’s baseline HR, which is considered as the resting or normal HR. On the other hand, the threshold for the RMSSD feature was calculated as the median of the RMSSD values computed during the entire baseline period, in order to make it more robust to outliers and work with non-normal distribution (Altman and Bland, 1983; Rossi, 2022).

The utilization of individualized thresholds allows for a relative comparison of the features, as it considers the baseline HR specific to each subject. Specifically, when comparing the Mean HR feature with its threshold, a 30% increase above the personalized threshold was employed. Likewise, for the RMSSD feature, a 50% decrease below the subject’s personalized threshold was used. This relative difference approach offers a more meaningful indication of significant changes in physiological responses, as it considers the unique baseline characteristics of each individual (Alexandre et al., 2012).

These two features are used in the analysis that compares the distress detection capabilities of E4 and Faros. The following steps were performed to compare the HR signals and their performance in detecting distress during each scene, so each step was performed on both E4 and Faros data, for each subject and each scene:

1. Threshold calculation: based on the Baseline measurement, calculated for each subject and its features.
2. Segmentation: HR signal is divided into non-overlapping segments of 10 s.
3. Feature calculation: Mean HR and RMSSD were calculated for each segment of the HR signal.
4. Threshold comparison: For each HR signal segment, both Mean HR and RMSSD are compared to their respective thresholds.
5. Distress detection: If the Mean HR of a segment exceeded its baseline threshold by 30%, it was considered elevated and labeled as one in the output vector. Similarly, if the RMSSD of a window was 50% below its baseline threshold, it was considered low and labeled as one in the output vector. Otherwise, a value of 0 was assigned, in both cases.
6. Threshold comparison vectors: for both Mean HR and RMSSD a threshold comparison vector was obtained.
7. Interpretation: A value of one in either the Mean HR and RMSSD threshold comparison vector pair indicated “medium distress level”, a value of one in both the Mean HR and RMSSD threshold comparison vector pair indicated “high distress level” and a value of 0 in both the Mean HR and RMSSD threshold comparison vector pair indicated “low distress level” or absence of it. This step was performed to granulate the data and present a more detailed distress state of a subject.

For example, if a Mean HR threshold comparison vector is equal to [0 1 0 1], and its RMSSD threshold comparison vector is equal to [1 1 0 0] the resulting distress vector would be equal to [1 2 0 1] which would translate to:

• 1: medium distress level
• 2: high distress level
• 0: low distress level
8. Distress level cases occurrence: number of occurrences for each level of distress was counted.

A statistical analysis was done to compare the occurrence of distress of a certain level (low, medium, or high) for IS, between Faros and E4. For this analysis, the Wilcoxon signed-rank test (Wilcoxon, 1992) was used, since it is suitable for comparing paired data from the same group if the data is not normally distributed. We have used it along with Cliff’s *delta* ( $C_{delta}$ ) as the effect size measure. In this case, the Faros and E4 data came from the same subjects, and it was measured during IS. The hypothesis related to this test is that there should be no statistically significant difference between the distress level occurrences detected by E4 and Faros during IS.

The test was conducted with a confidence level of 95%, ensuring a reliable measure of statistical significance. The obtained p-values were then compared to the predetermined alpha level of 0.05, allowing us to assess whether the observed differences were statistically significant.

#### 2.6.4 Interactive scene distress analysis

As it was already mentioned, during the interactive scene (IS) subjects are required to solve the Hanoi tower problem using a VR controller while being surprised by various distractors:

1. A box falls from the shelf in the 2nd min (120 s) after the scene starts.
2. The second box falls from the shelf in the 3rd minute (180 s) after the scene starts and the suspense sound effect is played.
3. The monster appears 4 min (240 s) into the scene, and it marks the end of the scene.

The idea of this analysis was to try to detect distress (if there is any) during the events at 120/180/240 s, for both Faros and E4. Since each HR signal is segmented into 10 s segments, the following steps are performed:

1. Segment extraction: For each event, three segments were extracted: (1) the segment immediately before the event, (2) the segment starting at the onset of the event with 10 s duration, and (3) the segment capturing the 10 s following the event onset. This approach accounted for potential delays in physiological responses while ensuring comprehensive coverage of distress reactions.
2. Distress detection was conducted by checking if distress was detected in at least one or two segments out of the three for each event situation. The resulting distress vectors explained in Section 2.4.4., step 7 was used to perform these checks.
3. Calculate distress occurrence in percents: based on the previous step values, the percentage of situations in which detected distress coincided with the VR events for each subject was calculated. This was done using two criteria: one-third (1/3) of the segments detecting distress and two-thirds (2/3) of the segments detecting distress. The resulting percentages ranged from 0% (no detected distress coinciding with VR events) to 100% (all detected distress coinciding with VR events).

## 3 Results

### 3.1 Data quality assessment results

**FIGURE 4:**
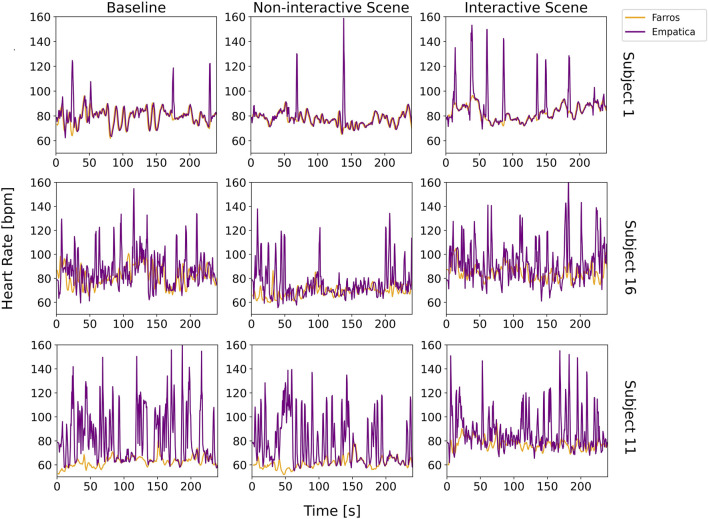
Visual comparison of Faros and E4 heart rate signals for three distinct cases (Subject 1, Subject 16, and Subject 11), for each scene.

To provide a comprehensive understanding of the data quality assessment, we start with a visual inspection of the HR signals from both devices. We specifically highlight three distinct cases, represented by Subjects 1, 11, and 16. These cases show case varying degrees of overlap between Faros and E4 signals: high overlap, medium overlap with E4 signal contamination, and low overlap with substantial E4 signal contamination, respectively (Figure 4). This visual representation serves as a foundational step in our analysis, allowing us to closely examine the specific differences in noise-related data quality assessment between the two devices.

In the case of Subject 1, there is little difference between Faros and E4 signals, for both scenes. However, occasional peaks in the E4 HR signal indicated the possibility of bad contact or unfiltered movement artifacts. Subject 16 exhibited a similar trend between the Faros and E4 HR signals. However, E4 signal was heavily contaminated with artifacts, likely caused by movement or other sources of unfiltered interference. For Subject 11, there was a clear lack of overlap between the Faros and E4 HR signals most of the time. The discrepancy could again be attributed to poor contact between the E4 device and the subject’s skin and/or data loss issues.

**FIGURE 5:**
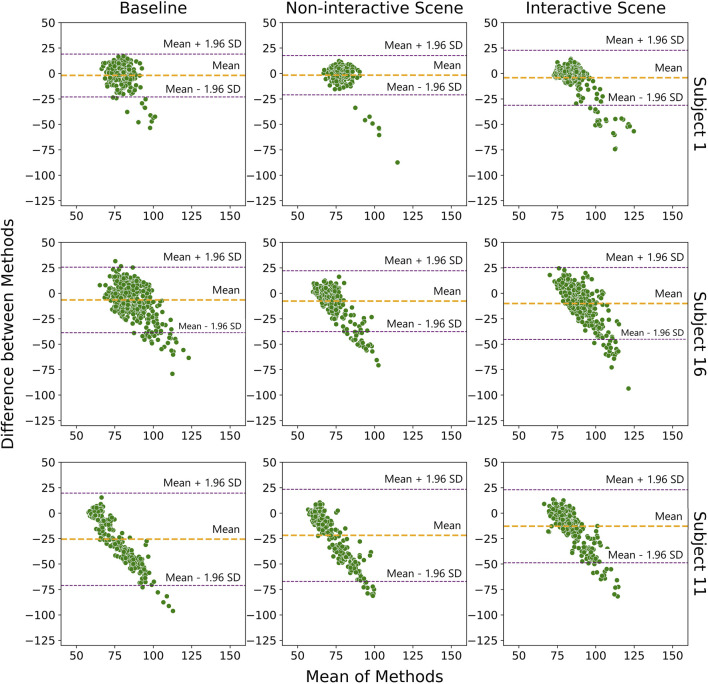
Bland-Altman plot showing the agreement between Faros and E4 heart rate signals for Subjects 1, 16, and 11. Orange line represents the mean difference in measurements between the two devices, and the two purple lines represent the upper/lower limit of the 95% confidence interval for the mean difference.

The Bland-Altman plot is visualized in Figure 5, and it implies similar conclusions as the ones obtained by observing visual comparison of Faros and E4 HR signals in Figure 4. This plot is often used to assess how similar a new instrument or technique is at measuring something compared to the instrument or technique used as the reference (Giavarina, 2015). The abscissa of the plot displays the average measurement of the two devices, and the ordinate displays the difference in HR measurements between E4 and Faros. The further the value of the average difference (orange horizontal line) is from zero, the larger the difference in measurements between the instruments.

Figure 5 shows that that value is further away from zero if we observe Subjects from top row to the bottom row, coinciding with Figure 4 and confirming that the measurement noise increases the average difference in measurements between E4 and Faros. Besides that, for Subject 1, most of the data points are inside the 95% confidence interval (purple horizontal lines), while for Subjects 16 and 11 we can observe that the data points are following a diagonal spread, indicating the discrepancy between measurements of E4 and Faros is biased proportionally to the magnitude of measurements. Also, in the upper right graph it can be seen that the measurements above the average line indicate biased comparison and are a consequence of subjects’ movements and more pronounced artifacts, as revealed in Figure 4.

Table 1 presents the mean and standard deviations of SNR values for each sensor and scene utilized in this study, calculated across all subjects. The results indicate that Faros consistently exhibited a higher mean SNR than E4 for all measurements.

**TABLE 1:** Signal-to-Noise Ratio (SNR) mean value and standard deviation calculated across all subjects, for both Faros and E4 and each scene.

Scene	Baseline		NIS		IS	
Sensor	E4	Faros	E4	Faros	E4	Faros
Mean ± SD [dB]	17.5 ± 3.2	22.4 ± 2.0	18.1 ± 3.9	24.2 ± 1.6	18.6 ± 3.3	22.9 ± 2.0

Furthermore, the standard deviations provide insights into the variability of the signal quality among the subjects for each sensor. Faros demonstrated smaller standard deviation values compared to E4, implying a greater degree of consistency in the SNR values across measurements.

Pearson and Spearman correlation coefficients mean values and standard deviation values, calculated between Faros and E4 across all subjects and for each scene, are displayed in Table 2. The correlation coefficients provide insights into the degree and direction of association between the data collected by the two devices.

**TABLE 2:** Pearson and Spearman correlation coefficients mean values and standard deviation calculated between Faros and E4 across all subjects, for each scene.

Scene	Baseline		NIS		IS	
Sensor	Pearson	Spearman	Pearson	Spearman	Pearson	Spearman
Mean ± SD	0.24 ± 0.32	0.30 ± 0.34	0.19 ± 0.24	0.27 ± 0.27	0.31 ± 0.27	0.36 ± 0.29

The results show that both Pearson and Spearman correlation coefficients consistently showed positive values for all scenes, indicating a positive linear relationship between the data captured by Faros and E4 sensors. The mean Pearson correlation coefficients ranged from 0.19 to 0.31, while the mean Spearman correlation coefficients ranged from 0.27 to 0.36. These positive correlation values suggest that as the physiological measurements from Faros increase, the measurements from E4 also tend to increase, and *vice versa*. However, these values do not indicate a strong correlation between the measurements, and that may be caused by the noise present in E4 measurements. Standard deviation values ranged from 0.24 to 0.34 for Pearson and from 0.27 to 0.29 for Spearman. These standard deviations represent the variability in the correlation values among the scenes.

The Mean Absolute Error (MAE) and Root Mean Square Error (RMSE) were computed to assess the agreement between the heart rate signals obtained from Faros and E4 devices for each subject and scene. The results presented in Table 3 indicate that for all three measurements (Baseline, NIS, and IS), the RMSE and MAE values were relatively small. For example, in NIS, the mean RMSE was 13.73 bpm, indicating an average difference of approximately 13.73 bpm between the HR measurements obtained from the two devices. The mean MAE in the same scenario was 8.87 bpm, representing an average absolute difference of around 8.87 bpm between the two measurements.

**TABLE 3:** Root Mean Square Error (RMSE) and Mean Absolute Error (MAE) mean values and standard deviation calculated between Faros and E4, across all subjects, for each scene.

Scene	Baseline		NIS		IS	
Sensor	RMSE	MAE	RMSE	MAE	RMSE	MAE
Mean ± SD [bpm]	14.4 ± 8.2	9.9 ± 6.5	13.7 ± 8.0	8.9 ± 6.0	13.2 ± 7.1	9.2 ± 5.9

Furthermore, the standard deviations of RMSE and MAE values were also reported for each scene, providing information about the variability in accuracy across different subjects. In general, smaller standard deviation values suggest a higher degree of consistency and reliability in the accuracy of heart rate measurements between Faros and E4 for individual subjects.

### 3.2 Distress detection using Faros and E4: intensity and frequency results

The results in Table 4 provide information on the frequency of distress events at different levels (low, medium, and high) during NIS and IS as captured by both Faros and E4 devices.

**TABLE 4:** Faros and E4 detected distress level occurrences for NIS and IS mean value and standard deviation.

Faros	NIS			IS		
	Low	Medium	High	Low	Medium	High
Mean ± SD [counts]	19 ± 8	3 ± 4	2 ± 6	9 ± 9	11 ± 7	7 ± 8

E4	NIS			IS		
	Low	Medium	High	Low	Medium	High
Mean ± SD [counts]	6 ± 8	14 ± 7	4 ± 5	1 ± 2	20 ± 6	6 ± 5

Table 4 shows that, on average, there were more high- and medium- and less low-level distress occurrences detected in IS than in NIS both when using Faros and E4 HR signal. Standard deviation was higher for Faros-detected distress occurrences compared to E4-detected distress occurrences for all cases except for medium-level distress in NIS where the trend was reversed.

Comparing the two devices, it can be observed that there are differences in the number of detected distress events in each category. For example, in NIS, E4 detected more medium-level distress events compared to Faros, while Faros recorded more low-level distress events. In IS, E4 detected noticeably more medium-level distress events compared to Faros, but a similar amount of high-level distress events.

**FIGURE 6:**
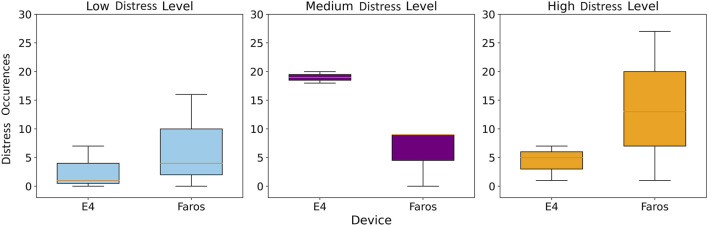
Boxplot comparisons of E4 and Faros for different distress level counts (left, middle, right plot) in IS.

The Wilcoxon signed-rank test comparing distress detection capabilities of both devices at all distress levels, is visually represented using boxplots in Figure 6 for IS, and the p-values for are displayed in Table 5.

**TABLE 5:** Distress intensity occurrences comparison between Faros and E4 device for different distress levels in IS. P-values were obtained using the Wilcoxon signed-rank test with 95% confidence interval.

Scene	Distress Intensity
Low	*p* < 0.01
Medium	*p* < 0.01
dHigh	*p* = 0.57

The *p* < 0.01 indicates that the difference in distress intensity occurrences between the devices is statistically significant for the Low and Medium distress levels. However, for the High distress level, the *p*-value of 0.57 suggests that there is no statistically significant difference in distress intensity occurrences between the devices. Cliff’s *delta* was calculated for comparison of distress occurrences of each level between Faros and Empatica, and it was equal to −0.652, 0.667 and −0.018 for Low, Medium and High distress occurrences, respectively. These values showed a significant difference between devices for Low and Medium distress occurrences, since any value above |0.474| is considered a large effect.

### 3.3 Interactive scene distress analysis results

The analysis of distress occurrence during the interactive IS was conducted using two criteria: detection in one-third (1/3) or two-thirds (2/3) of the segments (before, during, and after the triggering stimulus/event). The stricter criterion required distress presence for at least 20 s around the triggering event. The mean and standard deviation of the percentage of distress occurrences detected for both criteria (1/3 and 2/3) are shown in Table 6 for both Faros and E4 devices.

**TABLE 6:** Mean and Standard Deviation (SD) of percentage of distress occurrences detected coinciding with VR triggering situations during IS.

Device	Faros		E4	
Criteria	1/3	2/3	1/3	2/3
Mean ± SD [%]	88.9 ± 24.1	90.9± 21.6	98.2 ± 7.9	96.3 ± 15.7

The results indicate that both Faros and E4 devices detected a relatively high percentage of distress occurrences coinciding with triggering events during IS, with E4 showing slightly higher mean percentages compared to Faros. The standard deviation reflects the variability in event-related distress detection across participants for each criterion and device, showing that the results obtained using E4 HR signal were in a narrower range than the ones obtained using Faros HR signal (7.86% and 15.71% compared to 24.1% and 21.6%, respectively).

**FIGURE 7:**
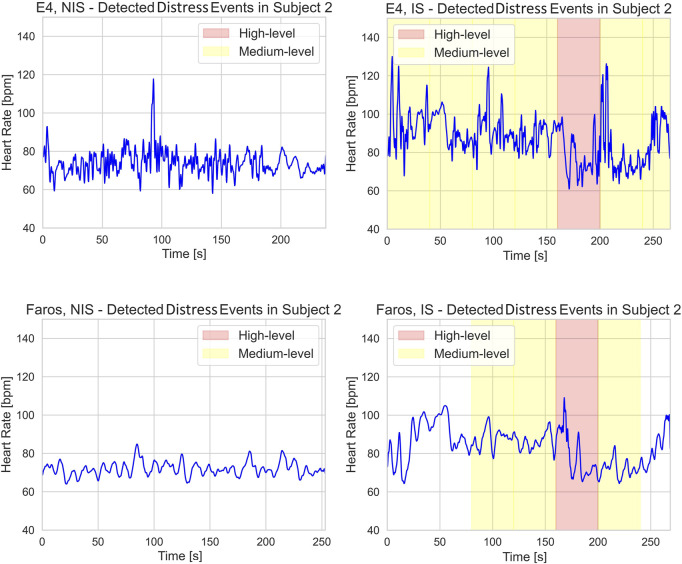
E4 (upper row) and Faros (lower row) heart rate signal with annotated medium- (yellow) and high- (red) level distress occurrences during NIS (left) and IS (right).

Figure 7 shows the periods of medium (yellow) and high (red) level distress occurrences for both E4 and Faros HR signals during NIS and IS. Although in NIS E4 HR signal is contaminated with more noise than Faros HR signal, there is no distress detected, which is expected since NIS is the scene without emotional events or triggers. The impact of noise on false positive distress occurrence detection can be seen in IS E4 HR signal, where Medium-level distress occurrence was detected from 0 to 80 s due to multiple high-intensity HR peaks that are not present in Faros HR signal.

## 4 Discussion

The presented study compared the distress detection capabilities of two wearable devices, Faros and E4. The results shed light on the strengths and limitations of each device and their potential applications in assessing emotional responses during interactive scenarios. Moreover, the analysis of distress occurrence during the interactive scene provided valuable insights into the physiological responses of participants during the dynamic and immersive virtual reality experience.

To explore if both devices used in this study can effectively detect distress during IS and answer the first research question, the analysis of distress occurrence was conducted, and it revealed interesting patterns during IS. Faros and E4 detected more high- and medium-level distress occurrences in IS than in NIS, demonstrating the impact of surprising elements on participants’ emotional responses. While E4 showed slightly higher mean percentages of distress occurrences compared to Faros, the devices exhibited similar overall performance in detecting distress occurrences during interactive scene-induced distress events. However, differences in detected distress events between two devices highlight the importance of considering device-specific factors in distress detection studies.

The Wilcoxon signed-rank test indicated that Faros and E4 exhibited statistically significant differences in distress intensity occurrences for low and medium levels, but not for high level in IS. Since the high distress level occurrences are the ones, we aimed to detect in the interactive scene, this is a significant result that indicates that both devices detected strong subject responses to distress inducing events, which implies that E4 could be used for high distress detection in motion-controlled environment. The significant differences between distress intensity occurrences for low and medium levels could be attributed to the noise contamination problem characteristic of E4 causing false positive distress detection. Noisy HR signal results in increased RMSSD values which prevents successful distress detection as with RMSSD we are looking for a decrease below the threshold. The significant difference between the two devices for low and medium distress levels is primarily the consequence of multiple false positives of E4 (cases where E4 detects distress even if it is not present). Since with E4 the RMSSD was less frequently below the designated threshold, therefore correctly interpreted as no distress, the occurrences of these false positive detections can mainly be attributed to the Mean HR signal of E4. This is not completely in line with the results from (Menghini et al., 2019) that clearly state that Mean HR measures for E4 show the best accuracy over various conditions. We believe the false positives observed in our study could also be attributed to the selected duration of the segments used for calculation as a 10 s segment is more prone to erroneous distress detection in case of a noisy HR signal.

Ragot et al. (2018) compared a laboratory based Biopac sensor to wearable E4 device for detecting emotion valence and intensity (distress) using selected features, for which the correlation coefficients ranged from 0.13 to 0.99, indicating non-consistency among different parameters. Our approach showed that higher distress levels are consistently detected with both devices when using appropriate feature engineering, comparable to the results of the Machine Learning approach used in (Ragot et al., 2018) on a similar-sized study sample of 19 volunteers. While valuable comparison presented in (Ragot et al., 2018) confirms that Empatica can be used for emotion valence and intensity classification in a non-noisy, static environment, our study shows that E4 device can be used for high-level distress detection in motion controlled, interactive VR environment.

With regards to our second research question, we addressed specifically the problem of data quality of E4 as this device is known to be more prone to motion artifacts and results in poorer SNRs. This was done mostly through noise contamination analysis and direct comparison of both devices. While Faros consistently exhibited higher mean SNR and smaller standard deviation, E4 signals occasionally showed false peaks indicating possible bad contact or unfiltered movement artifacts.

Both Pearson and Spearman correlation coefficients showed positive linear relationships between Faros and E4 data across all scenes. However, the correlation coefficients are relatively moderate, with large standard deviations, indicating that the strength of the association between the data from these two devices is not exceptionally strong. The moderate correlation can in our opinion again be explained by the motion (and other) artefacts, common for E4 measurements.

Despite relatively low correlation coefficients, small Mean Absolute Error (MAE) and Root Mean Square Error (RMSE) values indicate good agreement in measuring heart rate. The smaller standard deviation values for Faros further suggest higher consistency and reliability in accuracy compared to E4. It is important to consider that the Faros HR, obtained from the ECG signal with a higher sampling frequency of 500 Hz, provides better temporal resolution compared to E4 HR. This difference in resolution could also contribute to potential errors and discrepancies in the E4 HR signal.

It becomes evident that the presence of artifacts, poor contacts, data loss and different sampling frequency impact the reliability and alignment between the Faros and E4 HR signals. This corresponds to findings reported in (Schuurmans et al., 2020) where E4 also proved to be a practical and valid tool for research on HR and HRV, but only in movement-controlled conditions (in this study, subjects were meditating). With that, our third research question can be answered by stating that E4 device exhibits good performance in measuring heart rate, but with lower reliability and accuracy compared to Faros, due to the limitations based on lower sampling frequency and presence of artifacts and poor contacts that introduce noise to the measurement.

The results of our study and their interpretation should be considered with several limitations:

1. The noise present in the E4 HR signal after preprocessing still impacts the accuracy of distress detection, as observed in the example of Subject 2, IS with false positives. Future work should include the employment of advanced signal processing techniques to recognize noisy segments and to minimize the impact of noise on distress analysis.
2. We did not assess Heart Rate Variability (HRV) from Faros, as it may differ from Pulse Rate Variability (PRV) from E4 and thus could introduce even more discrepancies in results (Park et al., 2022).
3. The study focused on specific triggering events during IS to assess distress occurrences. While these events were carefully designed, they may not fully represent the complexity and variability of emotional responses in real-world scenarios. Further investigations incorporating a wider range of emotional stimuli and experiences would provide a more comprehensive understanding of distress detection in dynamic environments.
4. The main limitation of this study was the sample size, which was relatively small and age cohort as it included only university students and consisted of 8 females (6, since for two females the measurements were corrupted) and 10 males. This may limit the generalizability of the findings. Future studies with larger and more diverse samples are needed to validate the observed trends.
5. The study focused on distress intensity occurrences, which can be influenced by sex, age, individual differences and contextual factors. Incorporating these factors in future research would contribute to a more nuanced understanding of the complex interplay between physiological responses, individual differences, and emotional experiences. This includes intra-individual factors such as caffeine intake and fatigue levels, which may influence physiological responses and heart rate variability. We should consider controlling these variables in the future, for their potential impact on distress detection in VR settings.
6. We have mostly focused on the participant comfort and non-intrusivity of the setup, which is why we didn’t include measurement of signals like EEG, which require additional equipment, adding bulk and pressure and reduces participant comfort, but, for example, has been shown useful in classifying distress and no distress situations (Eldeeb et al., 2021). We should aim to assess additional physiological measurements that could provide more insights into the physiology behind the distress assessment using VR in healthy subjects.
7. No self-reported measures of distress were included in our study. While validated questionnaires could provide valuable ground truth data, they are inherently limited by biases such as social desirability, recall errors, acquiescence, and participant fatigue. Future studies should incorporate these measures, such as the Generalized Anxiety Disorder-7 (GAD-7) questionnaire (Spitzer et al., 2006), while carefully considering their limitations when interpreting self-reported data alongside physiological measurements

In this paper we presented a comprehensive approach to measuring and understanding subjects’ physiological responses within the motion-controlled VR environment by using two commercially available wearable devices. The study highlights primarily the importance of considering device-specific factors and data quality when using such wearable devices for distress detection. Faros demonstrated superior signal quality and consistency compared to E4 by retaining higher mean signal-to-noise ratios (from 4.3 dB to 6.1 dB) for all scenes, making it a more reliable choice for studies requiring high-quality HR data. Although correlation coefficients between data measured by Faros and E4 were consistently positive, they revealed relatively weak correlations with correlation coefficients below 0.4. Both devices, however, showed good agreement in measuring heart rate with average absolute difference less than 9 bpm, indicating their potential utility in assessing emotional responses during motion controlled interactive VR scenarios. Moreover, both devices performed well in detecting distress occurrences related to the triggering events and to the high distress levels. We found no statistically significant difference between Faros and E4 data for comparing high distress intensity occurrences (*p*-value = 0.57), while this is not true for low and medium distress intensities (*p*-value <0.01).

In addition to device comparison, we have also proposed a simple rule- and subject-specific threshold-based distress detection method that showed promising results and performance, especially when detecting distress coinciding with the distress-inducing events which were included in the interactive scene by design. Threshold fine-tuning and exploring different options and threshold values is out of scope of this paper, but it is one of the directions we would consider in our future work.

While E4 device shows promising potential as a practical alternative to Faros for distress detection, especially in scenarios where wrist-worn monitoring is preferred, researchers must be mindful of the specific research objectives and the level of data accuracy and consistency required. For studies that demand the highest level of data reliability and signal stability, Faros remains the preferred choice. Nonetheless, these findings open the door for further investigations and advancements in wearable physiological monitoring technologies. Our future research could include adding more distress-inducing scenarios and improving existing ones, considering more physiological features for distress detection, testing multiple commercially available devices, and trying to minimize the movement artifacts through device placement or different movement artifact removal methods. Future research could be directed towards examination of different distress inducing scenarios, comparison of other relevant physiological features for distress detection, testing multiple wearable devices, minimization of the movement artifacts with appropriate processing methods, and fine-tuning the feature thresholds for distress detection.

## Data Availability

The raw data supporting the conclusions of this article will be made available by the authors, without undue reservation.
