# Supplementary material for: Distress detection in VR environment using Empatica E4 wristband and Bittium Faros 360
Source: Front Physiol. 2025 Mar 5;16:1480018. doi: 10.3389/fphys.2025.1480018 (PMC11919861; doi:10.3389/fphys.2025.1480018)
Supplement: Supplementary file 1 [file Table1.docx]

Supplementary Material

# Supplementary Figures and Tables

## Supplementary Figures


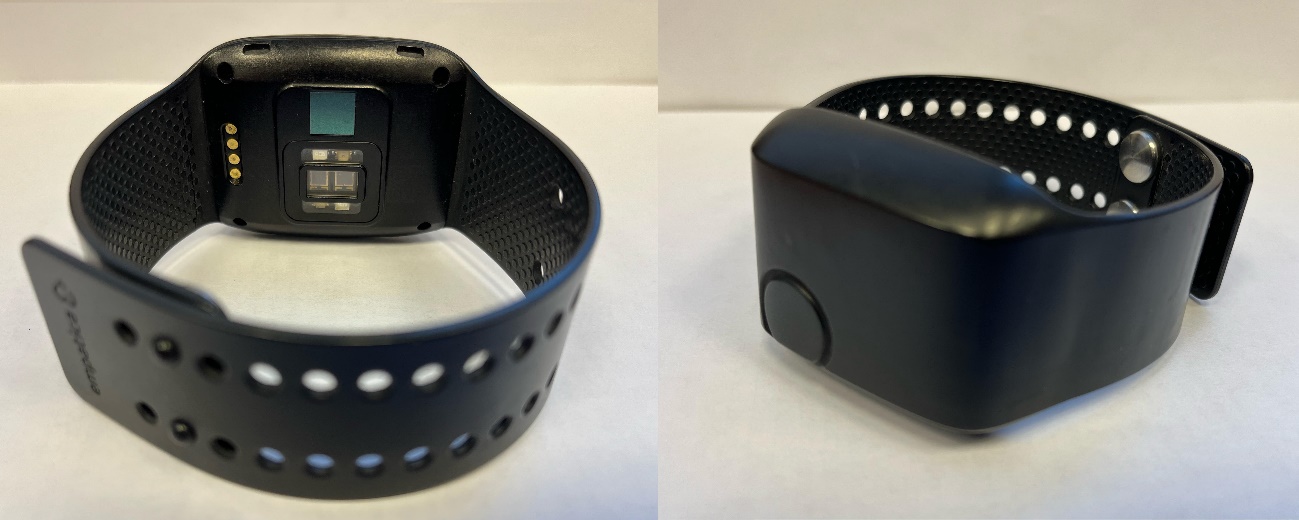


**Supplementary Figure 1.** Empatica E4 device. This photography was taken at the Faculty of Electrical Engineering, University of Ljubljana.

**
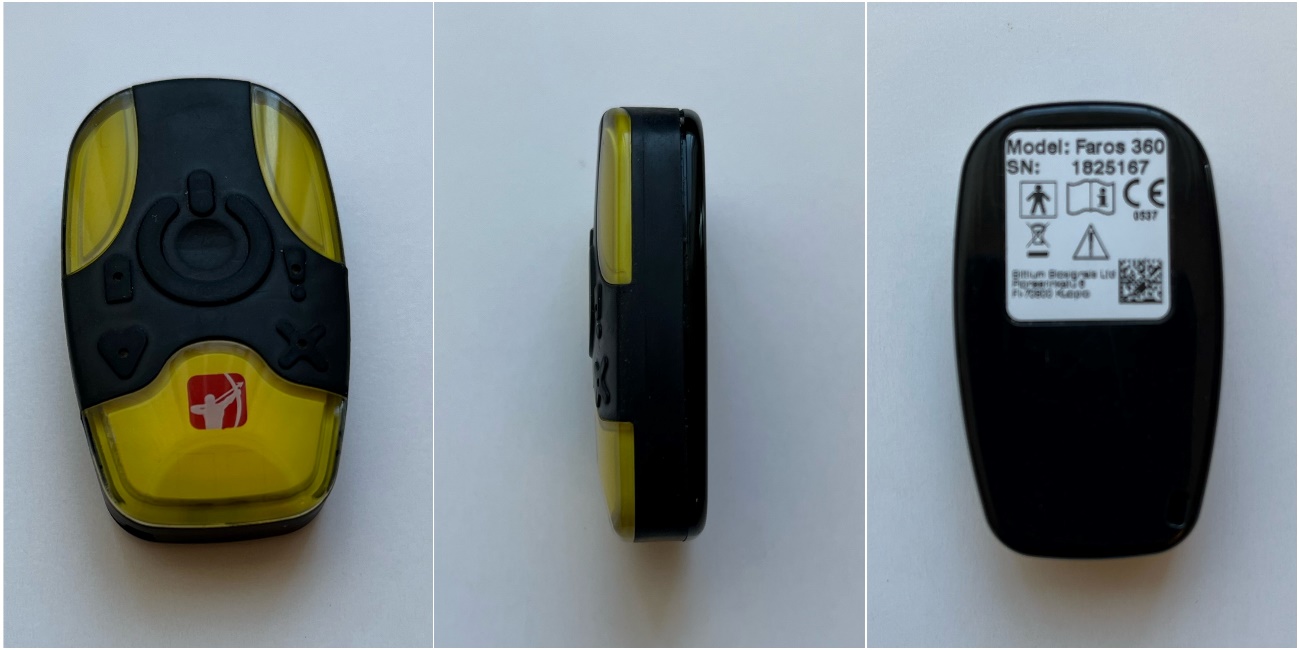
**

**Supplementary Figure 2.** Bittium Faros 360 device. This photography was taken at the Faculty of Electrical Engineering, University of Ljubljana.

**
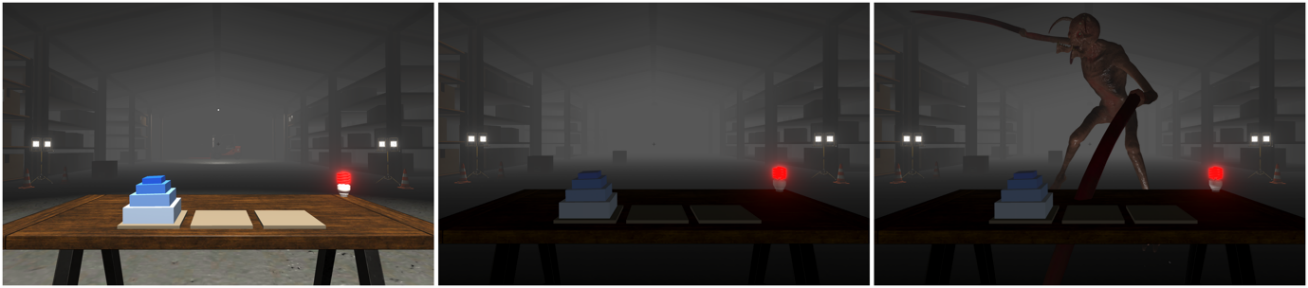
**

**Supplementary Figure 3.** left) Fall of the first box, center) fall of the second box, right) monster attack.

**
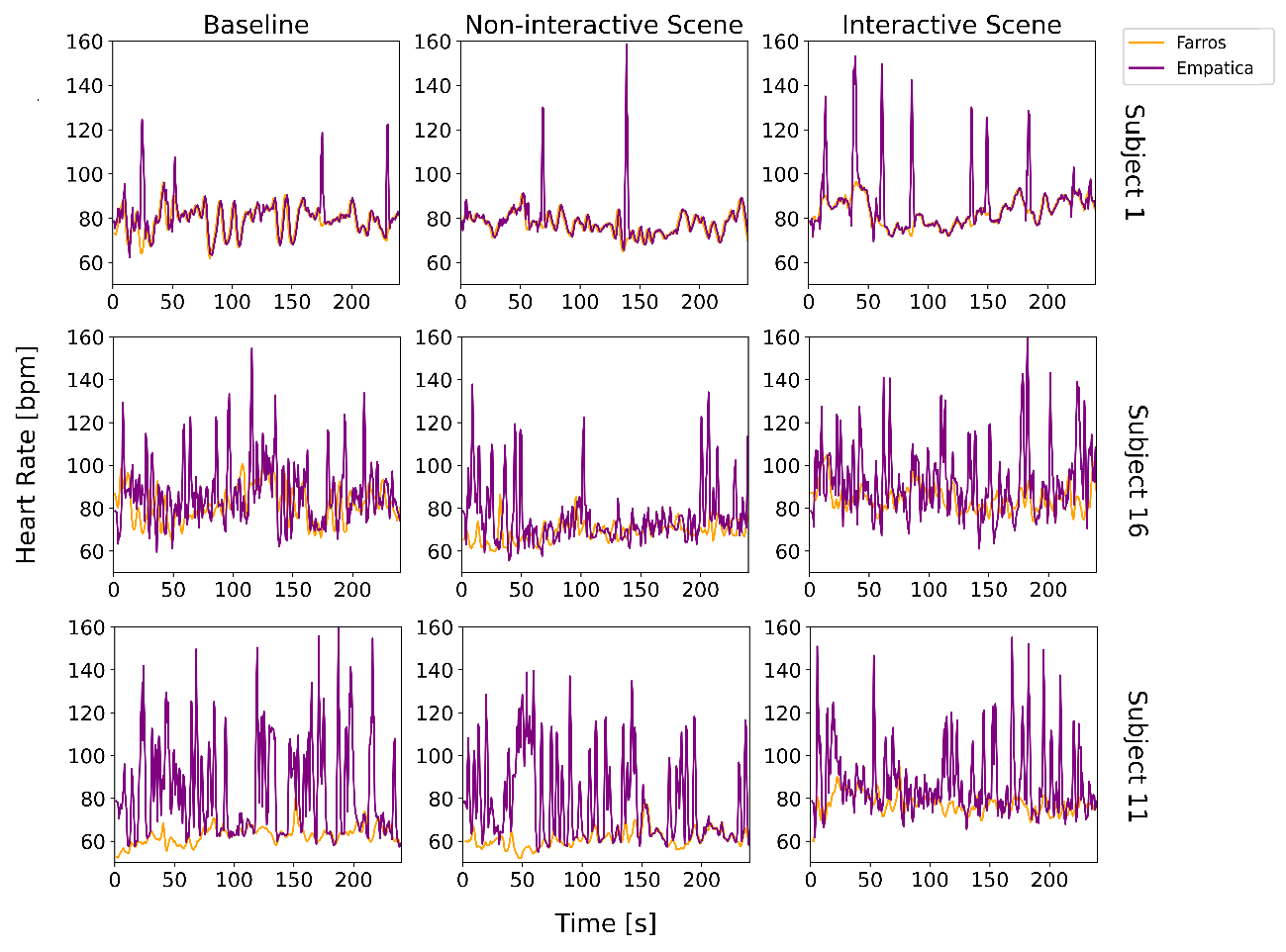
**

**Supplementary Figure 4.** Visual comparison of Faros and E4 heart rate signals for three distinct cases (Subject 1, Subject 16, and Subject 11), for each scene.

**
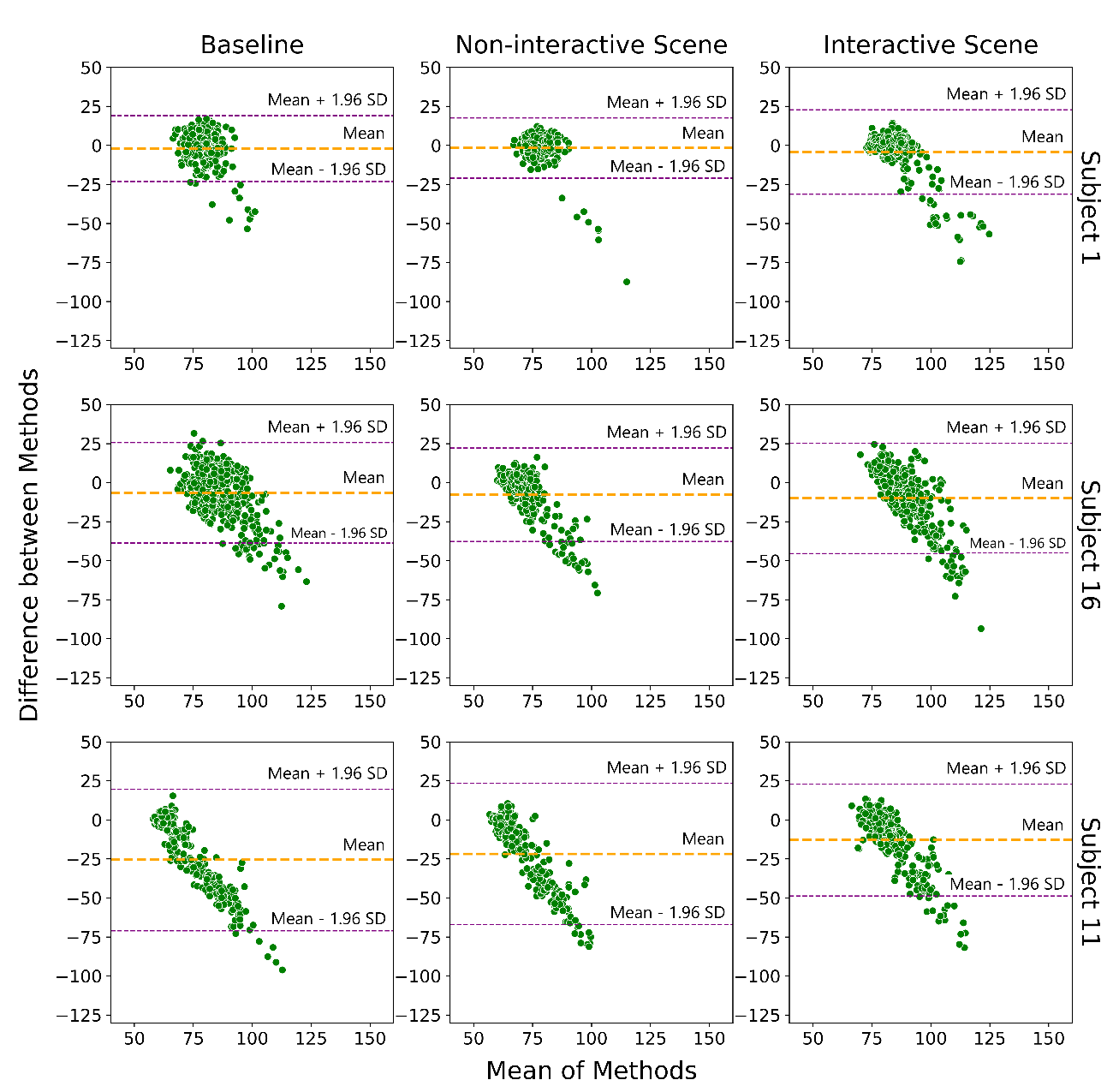
**

**Supplementary Figure 5.** Bland-Altman plot showing the agreement between Faros and E4 heart rate signals for Subjects 1, 16, and 11. Orange line represents the mean difference in measurements between the two devices, and the two purple lines represent the upper/lower limit of the 95% confidence interval for the mean difference.

**
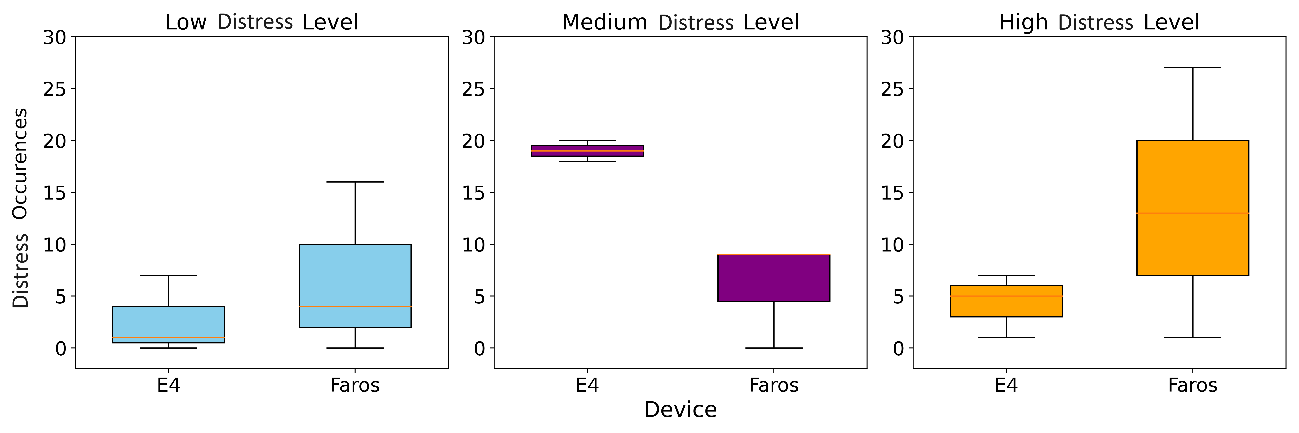
**

**Supplementary Figure 6.** Boxplot comparisons of E4 and Faros for different distress level counts (left, middle, right plot) in IS.

Faros (lower row).

**
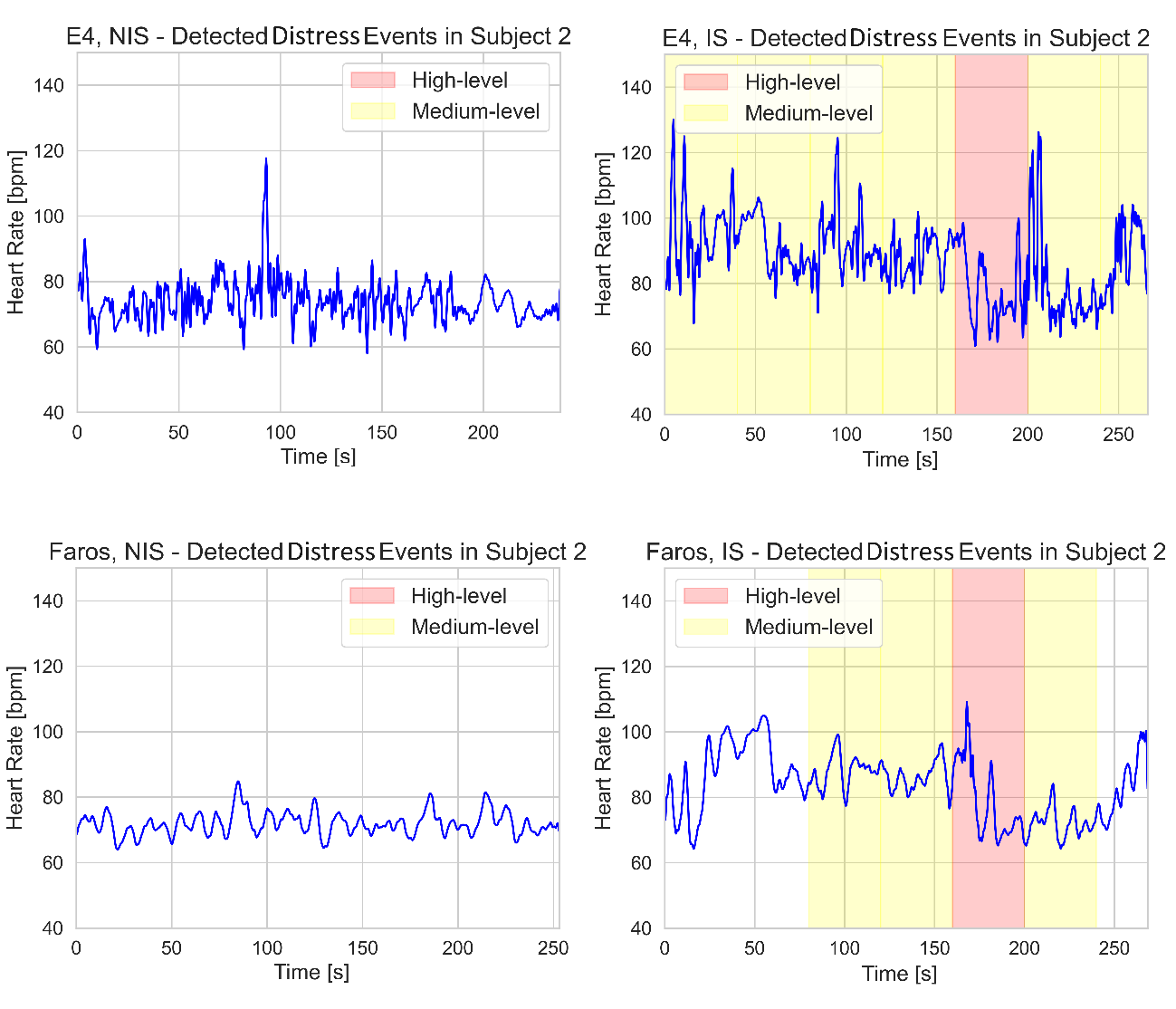
**

**Supplementary Figure 7.** E4 (upper row) and Faros (lower row) heart rate signal with annotated medium- (yellow) and high- (red) level distress occurrences during NIS (left) and IS (right).

## Supplementary Tables

| **Scene** | **Baseline** | | **NIS** | | | **IS** | | |
| --- | --- | --- | --- | --- | --- | --- | --- | --- |
| **Sensor** | **E4** | **Faros** | **E4** | **Faros** | **E4** | | **Faros** |  |
| **Mean ± SD [dB]** | 17.5 ± 3.2 | 22.4 ± 2.0 | 18.1 ± 3.9 | 24.2 ± 1.6 | 18.6 ± 3.3 | | 22.9 ± 2.0 |  |

**Supplementary Table 1.** Signal-to-Noise Ratio (SNR) mean value and standard deviation calculated across all subjects, for both Faros and E4 and each scene.

| **Scene** | **Baseline** | | **NIS** | | **IS** | |
| --- | --- | --- | --- | --- | --- | --- |
| **Sensor** | **Pearson** | **Spearman** | **Pearson** | **Spearman** | **Pearson** | **Spearman** |
| **Mean ± SD** | 0.24 ± 0.32 | 0.30 ± 0.34 | 0.19 ± 0.24 | 0.27 ± 0.27 | 0.31 ± 0.27 | 0.36 ± 0.29 |

**Supplementary Table 2.** Pearson and Spearman correlation coefficients mean values and standard deviation calculated between Faros and E4 across all subjects, for each scene.

| **Scene** | **Baseline** | | | **NIS** | | | **IS** | | |
| --- | --- | --- | --- | --- | --- | --- | --- | --- | --- |
| **Sensor** | **RMSE** | **MAE** | **RMSE** | | **MAE** | **RMSE** | | **MAE** |  |
| **Mean ± SD [bpm]** | 14.4 ± 8.2 | 9.9 ± 6.5 | 13.7 ± 8.0 | | 8.9 ± 6.0 | 13.2 ± 7.1 | | 9.2 ± 5.9 |  |

**Supplementary Table 3.** Root Mean Square Error (RMSE) and Mean Absolute Error (MAE) mean values and standard deviation calculated between Faros and E4, across all subjects, for each scene.

| **Faros** | **NIS** | | | **IS** | | |  |
| --- | --- | --- | --- | --- | --- | --- | --- |
|  | **Low** | **Medium** | **High** | **Low** | **Medium** | **High** | |
| **Mean ± SD [counts]** | 19 ± 8 | 3 ± 4 | 2 ± 6 | 9 ± 9 | 11 ± 7 | 7 ± 8 | |
| **E4** | **NIS** | | | **IS** | | | |
|  | **Low** | **Medium** | **High** | **Low** | **Medium** | **High** | |
| **Mean ± SD [counts]** | 6 ± 8 | 14 ± 7 | 4 ± 5 | 1 ± 2 | 20 ± 6 | 6 ± 5 | |

**Supplementary Table 4.** Faros and E4 detected distress level occurrences for NIS and IS mean value and standard deviation.

| **Scene** | **Distress Intensity** |
| --- | --- |
| **Low** | *p* < 0.01 |
| **Medium** | *p* < 0.01 |
| **dHigh** | *p* = 0.57 |

**Supplementary Table 5.** Distress intensity occurrences comparison between Faros and E4 device for different distress levels in IS. P-values were obtained using the Wilcoxon signed-rank test with 95% confidence interval.

| **Device** | **Faros** | | **E4** | |
| --- | --- | --- | --- | --- |
| **Criteria** | **1/3** | **2/3** | **1/3** | **2/3** |
| **Mean ± SD [%]** | 88.9 ± 24.1 | 90.9± 21.6 | 98.2 ± 7.9 | 96.3 ± 15.7 |

**Supplementary Table 6.** Mean and Standard Deviation (SD) of percentage of distress occurrences detected coinciding with VR triggering situations during IS.
